# Supplementary material for: From challenge to growth: A qualitative study of parental adaptation to Autism Spectrum Disorder
Source: PLoS One. 2026 Mar 17;21(3):e0345020. doi: 10.1371/journal.pone.0345020 (PMC12994777; doi:10.1371/journal.pone.0345020)
Supplement: S1 Table — (DOCX) [file pone.0345020.s001.docx]

**S1 Table. Codebook.**

| **Theme** | **Sub-Theme** | **Code** | | | **Description** | | |  |
| --- | --- | --- | --- | --- | --- | --- | --- | --- |
| **Family and Child Improvement** |  |  | | |  | | |  |
|  | **Family Empowerment** | Improvement _reducing emotional burden | | | Parent reports perceiving a decrease in the emotional burden associated with ASD | | |  |
|  |  | Improvement _autism literacy | | | Parent reports having developed greater awareness and ability to better understand ASD | | |  |
|  |  | Improvement _quality of life | | | Parent reports an overall improvement in their quality of life over time | | |  |
|  |  | Improvement _time for yourself | | | Parent reports having free time outside the family and being able to manage work and family commitments better | | |  |
|  | **Child Progress** | Improvement _autonomy | | | The parent reports an improvement in the child’s autonomy and independence | | |  |
|  |  | Improvement _communication skills | | | Parent describes child’s communication skills development (e.g., transition from nonverbal to verbal communication) | | |  |
|  |  | Improvement _behaviour | | | Parent reports significant improvements in child’s behaviour over time | | |  |
|  | |  | Improvement_ overall functioning | | | The parent reports significant improvements in the child’s overall functioning (e.g., child is described as more capable or independent) | | |
|  | |  | Improvement _social dimension | | | Parent reports significant improvements in social skills of the child over time | | |
| **Personal Growth and Trasformation** | |  |  | | |  | | |
|  |  | Acceptance and overcoming of critical phase | | | Parent reports having overcome the worst and/or are re-evaluating the overall meaning and impact of the diagnosis | | |  |
|  |  | Recognition of the child’s uniqueness | | | Parent expresses unconditional acceptance and love for their child, recognising their uniqueness | | |  |
|  |  | Growth of personal values | | | Parent reports a renewed connection with spirituality or a positive revision of life priorities or value system | | |  |
|  |  | Hope for the future/new possibilities | | | Parent expresses or describes a more optimistic and open view of the future, recognising new opportunities and potential for themselves and/or their child | | |  |
|  |  | Personal strength | | | Parent reports an increase in self-confidence and/or greater awareness of their own personal resources | | |  |
|  |  | Enhanced interpersonal relationships | | | Parent reports having more authentic and intimate relationships with others following the ASD experience | | |  |
| **Contextual Barriers** |  |  | | |  | | |  |
|  | **Problems with Care and Educational Services** | Traumatic diagnosis disclosure | | | Parent defines the communication of the diagnosis as traumatic | | |  |
|  |  | Delay in diagnosis | | | Parent reports having had difficulty obtaining an ASD diagnosis (delay in diagnosis) | | |  |
|  |  | Barriers in public care system | | | Parent reports difficulties in accessing adequate rehabilitation programmes and dissatisfaction with the public services they have contacted | | |  |
|  |  | Barriers in inclusive education | | | Parent reports critical issues in their experience with school services | | |  |
|  | **Family Issues** | Conflicts with partner | | | Parent reports relationship difficulties with their partner that have impacted on the management of the situation | | |  |
|  |  | Child’s autism-related challenges | | | Parent describes difficulties related to their child’s autism. | | |  |
|  |  | Lack of support from family of origin | | | Parent reports poor support or understanding from their family of origin | | |  |
|  |  | Partner’s denial of diagnosis | | | Parent reports that the partner has had difficulty accepting the child’s ASD | | |  |
|  | **Non-Autism- Friendly Environment** | Social exclusion | | | Parent reports having experienced exclusion of their child | | |  |
|  |  | Lack of local support/welfare | | | Parent reports poor welfare support (public services, government assistance) as a critical factor | | |  |
|  |  | Limited knowledge of ASD | | | Parent reports perceiving a lack of understanding of autism in the social context or fearing that the context would be unable to understand the situation due to a lack of knowledge about ASD | | |  |
| **Personal Barriers** |  |  | | |  | | |  |
|  | **Negative Emotional Experiences** | Caregiving-related fatigue | | | Parent reports fatigue and pressure related to the need for continuous care required by the child | | |  |
|  |  | Shame/stigma | | | Parent reports feeling or having felt fear of social judgement or feelings of isolation | | |  |
|  | **Maladaptive Coping Strategies** | Rumination | | | Parent reports recurring thoughts and focus on the negative aspects of the situation and/or thoughts and resources completely and obsessively directed towards managing the child | | |  |
|  |  | Avoidance | | | Parent reports having engaged (or currently engaging) in behaviours indicating non-acceptance (e.g. delay in requesting intervention; non-acceptance of the diagnosis) | | |  |
|  |  | Lack of flexibility | | | Parent expresses a lack of ability and flexibility in adapting educational strategies to the characteristics and needs of the child | | |  |
|  |  | Delegation of child’s care | | | Parent reports delegating intervention on their child to the services as experts and being reluctant to be involved in treatment | | |  |
|  | **Low Self- Efficacy in Parenting** | Low self-efficacy in parenting | | | Parent reports feeling inadequate in managing the complexities of care (e.g., difficulty communicating with their child, understanding their needs, and responding effectively to behavioural challenges and crises) | | |  |
| **Facilitating Contextual Factors** |  |  | | |  | | |  |
|  | **Resources for Managing and Understanding ASD** | Communication aids | | | Parent reports that tools aimed at improving communication with their child have played an important role in managing/coping with the situation | | |  |
|  |  | Access to information on ASD | | | Parent reports that access to information about the characteristics/management of ASD is a factor that has facilitated management and adaptation | | |  |
|  | **Meaningful Relationships and Social Context** | Inclusion | | | Parents report that their child’s social and educational inclusion has played or continues to play an important role in managing the situation | | |  |
|  |  | Support from social network | | | Parent reports having had (or currently having) support from friends, colleagues, etc. | | |  |
|  |  | Peer | | | Parents identify sharing experiences/difficulties with other parents of children with ASD (through informal networks and/or spontaneous contacts) as a key element of support | | |  |
|  | **Educational and Professional Resources** | Parental involvement in intervention | | | Parent reports that involvement in the interventions was a facilitating/supporting factor | | |  |
|  |  | Specialized services | | | Parent describes the positive role played by services, therapy centres or intervention programmes for adaptation/management | | |  |
|  |  | Schools | | | Parent reports positive and facilitating experiences with the school | | |  |
|  |  | Professional support | | | Parent values the presence and role of professionals in supporting their adaptation/management process | | |  |
|  |  | Parent training | | | Parent reports that parent training is/has been helpful in managing and/or adapting to the condition | | |  |
|  | **Family Resources** | Maternal initiative and advocacy | | | Parent (father) recognises that the active involvement and problem-solving skills of mothers are/have been a strength | | |  |
|  |  | Sibling as a source of support | | | Parent reports that the presence of other children in the family has played/plays an important role in adaptation/management | | |  |
|  |  | Couple cohesion and mutual support | | | Parent reports being able to rely on the support of their partner and/or perceiving this support as a resource | | |  |
|  |  | Support from family of origin | | | Parent reports receiving practical or emotional help from grandparents or other family members | | |  |
|  |  | Child’s strengths | | | Parent reports that the characteristics/behaviours of their child with ASD have facilitated management and adaptation | | |  |
| **Personal Strategies and Resources** |  |  | | |  | | |  |
|  | **Self-Efficacy** | Self- efficacy | | | Parent expresses a sense of confidence and mastery in interacting with their child, in their parenting role and/or in managing ASD | | |  |
|  | **Emotional and Reflective Coping Strategies** | Managing negative emotion | | | Parent reports adopting strategies aimed at effectively managing negative emotional states | | |  |
|  |  | Time for self-care | | | Parent reports engaging in self-care activities (e.g., exercise, social interactions, or spending time alone) | | |  |
|  |  | Deliberate rumination | | | Parent reports adopting or having adopted a form of targeted cognitive processing focused on making sense of their child’s condition | | |  |
|  |  | Disclosure | | | Parent reports openly discussing and disclosing their child’s ASD diagnosis as a coping and management strategy | | |  |
|  | **Agency** | Active problem-solving coping | | | | Parent reports actively facing or having faced challenges related to ASD by taking a proactive role and making efforts to identify practical solutions | |  |
|  |  | Gradual exposure to social situations | | | | Parent reports using a strategy of gradual exposure to social contexts as a means of supporting their child’s adaptation | |  |
|  |  | Collaboration with specialists and services | | |  | | Parent declares his/her commitment to work with therapists, teachers and other professionals to improve the child’s condition |  |
|  |  | Trial and error strategy | | |  | | Parent declares that he/she is implementing (or has implemented) a process of trial and error in managing autism, with a view to continuously adjusting strategies |  |
|  |  | Adaptation of educational strategies | |  | | Parent reports modifying and adapting their educational approach flexibly to respond to the child’s needs | |  |
|  |  | Proactive in seeking support | |  | | Parent declares that he/she has sought/is seeking appropriate therapy or support for his/her child | |  |
|  |  | Active information-seeking |  | | | Parent declares that they are committed to seeking information about ASD with the aim of better understanding their child | |  |
|  |  | Seeking emotional support |  | | | Parent reports seeking emotional support or someone to share their emotional experiences with | |  |
| **Emotional Distress** |  |  | | |  | | |  |
|  |  | Frustration | | | | Parent reports feeling (in the present) frustration and/or difficulty in their relationship with their child | |  |
|  |  | Uncertainty about the future | | | | Parent expresses a lack of a defined plan in their life (living day to day) or little confidence in the future | |  |
|  |  | Psychological suffering | | | | Parent expresses or reveals suffering in the present moment | |  |
|  |  | Sense of otherness | | | | Parent reports feeling different from parents of typically developing children | |  |
|  |  | Non acceptance | | | | Parent declares or expresses non-acceptance of the diagnosis and/or situation | |  |
|  |  | Resignation | | | | Parent describes a simple habit regarding the child’s condition (and not an elaboration) | |  |
