# Supplementary material for: From challenge to growth: A qualitative study of parental adaptation to Autism Spectrum Disorder
Source: PLoS One. 2026 Mar 17;21(3):e0345020. doi: 10.1371/journal.pone.0345020 (PMC12994777; doi:10.1371/journal.pone.0345020)
Supplement: Attachment — Submitted filename: Response_letter_auresp_2.docx [file pone.0345020.s003.docx]

**Response to the Editor**

Dear Editor,
We sincerely thank you for your constructive comments and the opportunity to revise our manuscript. We have carefully addressed each point, as detailed below.

Editor Comment 1:

*“Please briefly summarize the analytic approach (codebook thematic analysis and Big-Q framework) to ensure clarity for readers with diverse methodological backgrounds.”*

Response:
We thank the Editor for this helpful suggestion. We have revised the manuscript to strengthen the methodological description by clearly outlining how the codebook approach to Thematic Analysis and the Big‑Q qualitative framework guided our analytic process. Specifically, we expanded the *Data Analysis* section (now found in lines 182–196).

Editor Comment 2:

*“Please ensure that the data availability statement clearly specifies the minimal dataset available and the conditions under which qualified researchers may request access to additional de-identified data, in line with PLOS ONE requirements.”*

Response:
We fully revised the *Data Availability Statement* to comply with PLOS ONE’s policy for studies involving sensitive qualitative human‑participant data.

Once again, we thank you for your thoughtful guidance, which has strengthened the clarity and transparency of our manuscript. We hope the revisions meet your expectations and remain available for any additional clarification.

Kind regards,
Alessandro Musetti
